# Disrupted Working Memory Circuitry in Adolescent Psychosis

**DOI:** 10.3389/fnhum.2017.00394

**Published:** 2017-08-08

**Authors:** Ariel Eckfeld, Katherine H. Karlsgodt, Kristen M. Haut, Peter Bachman, Maria Jalbrzikowski, Jamie Zinberg, Theo G. M. van Erp, Tyrone D. Cannon, Carrie E. Bearden

**Affiliations:** ^1^Department of Psychology, UCLA Los Angeles, CA, United States; ^2^Department of Psychiatry, Rush University Medical Center Chicago, IL, United States; ^3^Department of Psychiatry, University of Pittsburgh Pittsburgh, PA, United States; ^4^Semel Institute for Neuroscience and Human Behavior, UCLA Los Angeles, CA, United States; ^5^Department of Psychiatry and Human Behavior, University of California, Irvine Irvine, CA, United States; ^6^Departments of Psychology and Psychiatry, Yale University New Haven, CT, United States

**Keywords:** schizophrenia, connectivity, development, adolescence, working memory capacity

## Abstract

Individuals with schizophrenia (SZ) consistently show deficits in spatial working memory (WM) and associated atypical patterns of neural activity within key WM regions, including the dorsolateral prefrontal cortex (dlPFC) and parietal cortices. However, little research has focused on adolescent psychosis (AP) and potential age-associated disruptions of WM circuitry that may occur in youth with this severe form of illness. Here we utilized each subject’s individual spatial WM capacity to investigate task-based neural dysfunction in 17 patients with AP (16.58 ± 2.60 years old) as compared to 17 typically developing, demographically comparable adolescents (18.07 ± 3.26 years old). AP patients showed lower behavioral performance at higher WM loads and lower overall WM capacity compared to healthy controls. Whole-brain activation analyses revealed greater bilateral precentral and right postcentral activity in controls relative to AP patients, when controlling for individual WM capacity. Seed-based psychophysiological interaction (PPI) analyses revealed significantly greater co-activation between the left dlPFC and left frontal pole in controls relative to AP patients. Significant group-by-age interactions were observed in both whole-brain and PPI analyses, with AP patients showing atypically greater neural activity and stronger coupling between WM task activated brain regions as a function of increasing age. Additionally, AP patients demonstrated positive relationships between right dlPFC neural activity and task performance, but unlike healthy controls, failed to show associations between neural activity and out-of-scanner neurocognitive performance. Collectively, these findings are consistent with atypical WM-related functioning and disrupted developmental processes in youth with AP.

## Introduction

Schizophrenia (SZ) is considered a neurodevelopmental disorder of brain connectivity (Stephan et al., 2006, 2009; Fatemi and Folsom, 2009; Pettersson-Yeo et al., 2011; Fornito et al., 2012; Fitzsimmons et al., 2013) but few functional magnetic resonance imaging (fMRI) studies have examined brain connectivity during the putatively critical developmental period of adolescence. To date, the focus has been on connectivity abnormalities in adults with SZ by examining neural activity during cognitively demanding tasks, such as working memory (WM). Deficits in WM, particularly visuospatial, are a well-documented and robust core feature of SZ (Silver et al., 2003; Lee and Park, 2005; Piskulic et al., 2007; Forbes et al., 2009; Park and Gooding, 2014). Furthermore, WM impairment is considered a reliable cognitive endophenotype of SZ given the presence of WM deficits and related neural dysfunction in clinically unaffected relatives (Callicott et al., 2003a; Saperstein et al., 2006; Knowles et al., 2014) and individuals with elevated genetic and clinical risk (Glahn et al., 2003; Wood et al., 2003; Smith et al., 2006; Fusar-Poli et al., 2010; Choi et al., 2012). Deficits in WM have also been shown to predict future development of overt psychosis (Brewer et al., 2006; Pukrop et al., 2007).

Although visual short term capacity has been estimated at approximately four separate items among healthy individuals (Todd and Marois, 2004; Cowan, 2010), individual variability (Cowan, 2001, 2010; Gold et al., 2003; Barrett et al., 2004; Unsworth and Engle, 2007) has led to the estimation of subjects’ individual short-term WM capacity from behavioral data (Cowan, 2001). Individual capacity has been used to assess neural circuitry abnormalities in SZ, with patients demonstrating decreased individual visual WM capacity compared to healthy controls across a range of tasks; this has been posited to result from difficulties encoding the information (Gold et al., 2003, 2010; Jansma et al., 2004) and/or impaired attentional control (Mayer et al., 2012; Leonard et al., 2013). Spatial WM capacity among adults with SZ also correlates with overall cognitive abilities (e.g., IQ; Johnson et al., 2013).

Neuroimaging studies to date have largely focused on the dorsolateral prefrontal cortex (dlPFC) and parietal cortex, key regions involved in WM processing (e.g., Jonides et al., 1998; D’Esposito et al., 2000; Petrides, 2000; Curtis and D’Esposito, 2003; Constantinidis and Wang, 2004; Pasternak and Greenlee, 2005), though a larger network of WM-related dysfunction including the anterior cingulate cortex (ACC) and left frontal pole has also been proposed (Glahn et al., 2005). Specifically, dlPFC activity among SZ patients varies depending on task load demands and range of capacity/performance ability (Manoach, 2003; Jansma et al., 2004; Karlsgodt et al., 2009), suggesting generalized dlPFC “inefficiency” during WM (Potkin et al., 2009). Notably, these studies did not directly utilize capacity load estimates in group comparisons of neural activity during WM performance, focusing primarily on *post hoc* correlations and regressions. However, the proposed “neural inefficiency” in patients with SZ mimics the inverted-U pattern described among healthy individuals; while increased WM demand is associated with increased activity within the dlPFC and other regions (e.g., superior frontal cortex, intraparietal cortex; Klingberg et al., 2002; Curtis and D’Esposito, 2003; Finn et al., 2010), dlPFC activation decreases once WM load exceeds individual capacity (Callicott et al., 2003b; Manoach, 2003; Van Snellenberg et al., 2015). Additionally, reduced connectivity between fronto-parietal and fronto-hippocampal regions during WM performance among patients with SZ has been associated with severity of positive symptoms and reduced task accuracy in a cross-sectional analysis, in line with neural dysfunction underlying the clinical and cognitive phenotype (Henseler et al., 2010).

However, the focus on WM dysfunction among adults with SZ disregards the major neural reorganization that occurs in adolescence (Paus, 2005; Insel, 2010; Stiles and Jernigan, 2010; Petanjek et al., 2011). This is striking, as age-related increases in neural activity have been found within core fronto-parietal WM circuitry during visual WM tasks in healthy adolescents (Andre et al., 2016). Moreover, significant associations between WM capacity and neural activity have been found in the same regions, suggesting that WM capacity may also increase with age (Klingberg et al., 2002). Yet the literature remains inconsistent, as increasing age has also correlated with decreasing activation in the superior frontal, limbic cingulate gyrus (Andre et al., 2016), and superior parietal regions (Schweinsburg et al., 2005). Regardless, differences in the role of the PFC during WM performance can be distinguished within the adolescent period. For example, while the PFC is recruited during WM tasks throughout adolescence, neural activity correlates with behavior (i.e., task accuracy) only in late adolescence (Finn et al., 2010), suggesting further refinement of WM-related circuitry and PFC maturation with increasing age (Casey et al., 2005; Paus, 2005; Petanjek et al., 2011).

Given this role of age on neural and cognitive development, an investigation of WM deficits and underlying neural dysfunction among individuals who develop overt psychosis during adolescence may be particularly enlightening. Adolescent psychosis (AP; overt psychosis emergence prior to age 18) is a particularly virulent and chronic form of psychotic disorder that is associated with poor prognosis (Vyas and Gogtay, 2012). AP is also typically associated with more severe cognitive deficits relative to the adult-onset form of illness, particularly in the domain of WM (Frangou, 2010; Zabala et al., 2010). This model therefore may provide greater insight into the neural and neurocognitive abnormalities associated with the disorder, while simultaneously allowing for investigations of effects of earlier onset age on brain development.

Existing functional imaging studies of WM in AP have revealed both abnormal patterns of neural activity across brain regions critical for higher-order cognitive activity (e.g., frontal regions, ACC) and disrupted functional connectivity within prefrontal/limbic and visual processing networks (e.g., occipital lobe) relative to healthy controls (Thormodsen et al., 2011; White et al., 2011a,b; Kyriakopoulos et al., 2012; Sugranyes et al., 2012; Bittner et al., 2015). AP patients also evidence reduced coupling of the dlPFC with other key regions implicated in WM (e.g., ACC) as compared to healthy adolescents when individual capacity is not factored in Kyriakopoulos et al. (2012). Interestingly, an investigation of age-associated changes revealed decreases in dlPFC activity and increases in dlPFC-ACC coupling among AP patients as compared to controls, suggesting growing inefficiency of neural networks with increasing age (Kyriakopoulos et al., 2012). However, to our knowledge, only one prior study of AP to date has considered individual capacity, finding that relative to healthy controls, AP patients evidence reduced capacity at each WM load and a negative correlation between neural activity and capacity during a late maintenance phase (Bittner et al., 2015). Correspondingly, the literature addressing functional dysconnectivity during WM performance in AP is still in its infancy, particularly with respects to the effects of manipulating memory demand and accounting for individual WM capacity on task-based activation and functional connectivity. The utility of incorporating each subject’s capacity into analyses has been previously described for a verbal WM task (Karlsgodt et al., 2009). Briefly, this method attempts to control for differences in neural activity that might result when task demands exceed an individual’s own WM ability. Additionally, by more accurately capturing the WM-related neural activity/connectivity present at optimal capacity, inconsistencies in the literature may be resolved.

The present study therefore investigated behavioral correlates of neural activity and connectivity during WM engagement in adolescents with AP relative to typically developing controls. As a novel extension of prior work, we examined the relationship between individual WM capacity, calculated via a parametric manipulation of WM load, and task-based neural activation, and further assessed the association with development. In particular, we examined whether the fine-tuning of functional networks during adolescence is disrupted in AP, which may result in an absence of typical age-associated increases in focal activation as well as abnormal patterns of functional connectivity, particularly in the prefrontal and parietal regions. Lastly, we examined the relationship between WM-related neural dysfunction and out-of-scanner neurocognitive performance. We thus hypothesized the following:

1. Individuals with AP will evidence WM impairment compared to controls, as indexed by lower overall WM capacity and decreased task accuracy at higher spatial WM loads.
2. Controlling for individual WM capacity, AP patients will show an abnormal pattern of neural activity within WM-relevant neural circuitry (i.e., prefrontal and parietal cortices) during task performance relative to controls. Specifically, based on prior studies in adult patients with SZ, we expect youth with AP to evidence reduced neural activity in dlPFC and parietal regions, but increased activity in less task-relevant regions, such as the frontal pole, anterior cingulate and occipital cortex.
3. Relative to controls, AP patients will demonstrate reduced efficiency of WM-related neural circuitry as evidenced by a decoupling of typically interactive regions (e.g., fronto-parietal connections).
4. Given that patients with AP are hypothesized to differentially and/or inefficiently recruit relevant brain regions during WM performance as a function of increasing age, we anticipate that, relative to controls, AP patients will show an altered pattern of age-associated changes in WM circuitry. Specifically, patients will fail to show the expected positive association between age and increased neural activity within frontal and parietal regions during task performance.
5. Decreased neural activity during spatial WM task administration will be associated with poorer behavioral performance and poorer performance on neurocognitive tasks completed outside the scanner.

## Materials and Methods

### Participants

Twenty-one healthy volunteers (18.07 ± 3.26 years old, range = 14.81–21.33 years) and 23 AP patients (16.58 ± 2.60 years old, range = 13.98–19.18 years) were recruited as part of a larger, ongoing study (UCLA Adolescent Brain-Behavior Research Clinic; ABBRC). Demographic variables (age, IQ, participant and parental education level) did not differ between the groups, nor did gender, handedness and race/ethnicity distributions (see Table 1 for demographic and diagnostic information). AP patients with past substance abuse diagnoses were permitted to participate if they were free of substance abuse for the preceding 6 months; patients with substance dependence diagnoses were excluded. Inclusion criteria for AP patients included the following diagnoses: SZ, psychotic disorder not otherwise specified (NOS), schizophreniform disorder and schizoaffective disorder. All control participants were free of Axis I disorders and of SZ-spectrum disorders among first-degree relatives. This study was carried out in accordance with the recommendations of UCLA’s Institutional Review Board with written informed consent from all subjects, and from their parents for participants under the age of 18. All subjects gave written informed consent in accordance with the Declaration of Helsinki. The protocol was approved by the UCLA’s Institutional Review Board.

**Table 1:** Demographic information characterizing study sample^1^.

	Controls (*N* = 17)	AP patients (*N* = 17)	*p* value
Mean age, years (± SD)	18.07(3.26)	16.58(2.60)	0.15
[range, years]	[14.81–21.33]	[13.98–19.18]	
Number female (%)	8(47.1)	6(35.3)	0.49
Number left-hand dominant (%)	0(0)	1(5.9)	0.31
Mean participant education, years (± SD)	11.53(2.62)	10.41(2.29)	0.20
Mean parental education, years (± SD)	15.97(2.67)	14.59(2.45)	0.64
Race/Ethnicity (%)			0.61
Caucasian, Non-Hispanic	10(58.82)	9(52.94)	
Caucasian, Hispanic	2(11.76)	5(29.41)	
African-American	2(11.76)	1(5.88)	
Asian-American/Pacific Islander	2(11.76)	2(11.76)	
Other	1(5.88)	0(0)	
Diagnoses (%)			
Schizophrenia	0(0)	6(35.29)	
Psychotic disorder NOS	0(0)	5(29.41)	
Schizophreniform disorder	0(0)	3(17.65)	
Schizoaffective disorder	0(0)	3(17.65)	
Medication (%)^2^			
Atypical antipsychotic	0(0)	10(58.82)	
Typical antipsychotic	0(0)	1(5.88)	
SSRI	0(0)	6*(35.29)	
Mood stabilizer	0(0)	2^§^(11.76)	
Antidepressant	0(0)	3^§^(17.65)	
Anxiolytic	0(0)	3^§^(17.65)	
Sedative	0(0)	1^§^(5.88)	
Anticonvulsant	0(0)	2(11.76)	
Mean SIPS: total positive symptoms score (±SD)^3^	1.38(1.96)	16.40(7.20)	<0.001
Mean neurocognitive score (±SD)^4^			
WASI IQ	111.56(11.34)	103.00(14.92)	0.08
WMS spatial span	11.63(3.46)	9.50(3.25)	0.08
WAIS-III digit span	11.88(2.63)	9.50(3.46)	0.04
Mean load corresponding to highest capacity	3.76(0.44)	3.24(0.75)	0.02

^1^Mean values for each continuous variable were tested for group differences at the univariate level. Gender, handedness and race/ethnicity distributions were tested with *Chi*-squared analyses; no significant group differences were detected for any comparisons except on clinical and neurocognitive measures (all *p* > 0.05). ^2^Patients reported a mean of 94.15 (*SE* = 27.85) days on antipsychotic medication and a mean of 130.48 (*SE* = 41.04) days on other psychoactive medications at the time of assessment. Mean days on medication was missing for one patient. Medication history was missing for one adolescent psychosis (AP) participant and one control participant. ^3^SIPS, Structured Interview for Prodromal Syndromes. Higher scores denote better levels of functioning. SIPS data was missing for one control participant and seven AP participants. ^4^WASI, Wechsler Abbreviated Scale of Intelligence; WMS, Wechsler Memory Scales-3; WAIS-III, Wechsler Adult Intelligence Scale-III. Neurocognitive data was missing for one control and one AP patient. *Taken concurrently with antipsychotic medication for all but one patient. ^§^Taken concurrently with antipsychotic medication.

### Behavioral Assessments

All diagnostic and neuropsychological assessment measures used have been previously described (Bachman et al., 2012). Diagnoses for all participants were determined using the Structured Clinical Interview for DSM-IV Axis I diagnoses (SCID; First et al., 1998) and by review of medical records; final diagnoses required consensus among supervising clinical psychologists. Current level of symptomatology (within the current month of the clinical assessment) was determined via the Structured Interview for Prodromal Syndromes (SIPS; McGlashan et al., 2001). Participants were also administered a neurocognitive battery, including measures of intelligence (Wechsler Abbreviated Scale of Intelligence—Full-scale IQ, T-score) and WM (Wechsler Memory Scales-3 (WMS)—Spatial Span, total scaled score; Wechsler Adult Intelligence Scale-III (WAIS-III)—Digit Span, total scaled score). Control subjects were screened for Axis I disorders with the SCID and for history of SZ-spectrum disorders among first-degree relatives using the Family Interview for Genetic Studies (FIGS; Maxwell, 1992). All assessments were administered by clinicians trained to a standard reliability criterion (Ventura et al., 1998). Medication information was obtained via participant and parent/guardian report and medical record review.

### fMRI Acquisition and SCAP Task

Following behavioral assessments, participants were scanned on a 3.0 Tesla (3T) Siemens Allegra scanner. The fMRI sequence consisted of 180 echoplanar images for a total scan time of 9 min (TR/TE 3000/45 ms, 90° flip angle, 33 3 mm slices). While in the scanner, participants were administered a spatial WM task assessing spatial capacity (SCAP), which has been shown to be sensitive to spatial WM deficits in individuals with SZ (Glahn et al., 2003; Cannon et al., 2005). The SCAP task involved showing participants a target array of 1, 3, 5, or 7 yellow circles per trial (2-s presentation) after a 1-s fixation period. Following a fixed delay of 3 s, subjects were shown a probe of a single green circle for 3 s. They were then asked whether the probe dot’s location corresponded to a location of one of the yellow target dots in the most recently presented set. There were 12 trials of each load (48 trials in total) presented in two acquisition sessions. Each load was presented in pseudorandom order in sets of two trials (three per session), and data were analyzed in those blocks (correct trials only). To better isolate effects due to WM activity only, the fixation period was excluded from analysis. Preprocessing steps included the following: functional analysis was performed using FSL (FMRIB’s Software Library v3.3; Smith et al., 2004). Each BOLD image in the time series was registered (using a 3D co-registration, six parameter rigid-body) to the middle data point. Data were then registered, first the EPI to the subject’s individual T2-weighted structural image, then the T2 to the study specific common brain (Jenkinson and Smith, 2001; Jenkinson et al., 2002). Individual subject analyses employed FMRI Expert Analysis Tool (FEAT) using a 5 mm (FWHM) Gaussian smoothing kernel and 72 s high-pass filter. Time-series statistical analysis on each subject was carried out using FMRIB’s Improved Linear Model (FILM) with local autocorrelation correction (Woolrich et al., 2001). Regarding the design matrix, in the individual first-level analyses, loads 1, 3, 5 and 7 were modeled. Participants with more than 3 mm of average translational motion were also excluded from subsequent analyses (*n* = 6 patients, four controls), resulting in a final sample of 17 patients and 17 controls. Timepoints corresponding to motion outliers were added to the model as nuisance regressors using framewise displacement as determined by FSL motion outliers^1^. Analyses of overall neural activation utilized a whole-brain approach.

### Statistical Analysis

#### Analysis I: Behavioral Performance

Behavioral data from the SCAP task were analyzed in SPSS (v20) using repeated measures ANOVA with group (AP patients or controls) as the between subjects factor, load as the within subjects factor, and percent correct at each load as the dependent variable (as described in Karlsgodt et al., 2009; Shilyansky et al., 2010). Additionally, we covaried for age. Between-group differences in reaction time were also examined.

#### Analysis II: WM Capacity-Associated Neural Activity and Age-Associated Effects

In order to examine group differences in neural activity during SCAP task performance, each subject’s WM capacity was first calculated at each load. The formula k = n*(H + CR − 1) was used, where *k* = capacity, *n* = load #, *H* = hit rate and *CR* = correct rejection rate (Cowan, 2001). Final capacity was identified by the highest value calculated; the load corresponding to each subject’s highest capacity was entered into group analyses. Overall group differences in WM capacity (via selected load) were compared using SPSS (v20). Group analyses related to neural activity were then performed using FSL FEAT (Local Analysis of Mixed Effects; FLAME), which has been shown to be less vulnerable than other methodologies to inflation of familywise Type-1 error rates (Eklund et al., 2016), with age, gender and handedness as covariates. Overall behavioral performance (% correct) was also included as a covariate to control for differences in ability related to clinical status and to ensure group differences in magnitude of activation were not due to non-specific effects (e.g., effort or strategy; Meda et al., 2009; White et al., 2011a; Satterthwaite et al., 2013; Wadehra et al., 2013). Main effects of group and age were modeled, as well as a group-by-age interaction, in order to investigate differential effects of age between groups. Threshold for cluster statistical significance was set at *Z* > 2.3, *p* < 0.05, with multiple comparison correction implemented in FSL FEAT (Friston et al., 1994; Forman et al., 1995; Jenkinson and Smith, 2001).

#### Analysis III: Psychophysiological Interaction (PPI) Analysis and Age-Associated Effects on Functional Connectivity

**Figure 1:**
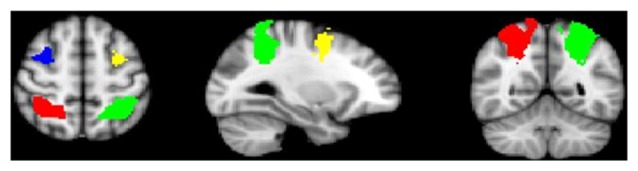
Anatomical-functional regions of interest (ROIs; bilateral dorsolateral prefrontal cortex (dlPFC) and parietal cortex).

To test whether patients show a de-coupling of regions that typically are functionally connected during WM demands (e.g., dlPFC with parietal regions), a psychophysiological interaction (PPI) analysis (O’Reilly et al., 2012) was conducted. Structural regions of interest (ROIs) including the dlPFC and parietal cortex in each hemisphere were identified using a probabilistic cluster atlas (Harvard-Oxford, 2 mm). Next, functional ROIs were defined in the study-specific average brain space. Activation clusters were identified using the FSL cluster option from the all-participants, all-loads omnibus contrast. Those that overlapped with the above anatomical ROIs were masked. Final masks were created from the voxels common to both the functional and anatomical ROIs, resulting in four final ROIs in the right and left dlPFC and parietal lobes (Figure 1). Mean activation for each ROI was then extracted following registration to each participant’s preprocessed data.

First-level PPI analyses modeled the interaction between mean activation within each ROI and load condition, with loads determined by each subject’s WM capacity as previously defined; other load conditions were controlled for. Group analyses were then modeled identically as described above, including main effects and group-by-age interaction. Specifically, the regressors used in the PPI analysis included age, gender, handedness and overall behavioral performance (% correct).

#### Analysis IV: Association between Neural Activity and Task Performance/Neurocognitive Measures

Partial correlations were calculated examining the relationship between neural activity within WM task-related regions (% signal change; %SC) within each bilateral dlPFC and parietal ROI) and task performance (% correct), controlling for the effects of age and gender. Similar partial correlations were performed for neural activity and each of three neurocognitive measures completed outside of the scanner (IQ, digit span, spatial span). IQ was particularly examined given previous findings that spatial WM capacity is associated with IQ among adults with SZ (Johnson et al., 2013). Due to the inherent group differences in task performance and neurocognition, correlations were run separately for AP patients and controls, with a total of 16 comparisons per group. Given the exploratory nature of these analyses, comparisons for multiple corrections were not performed. In order to determine %SC, the Featquery^2^ program applied the inverse of the initial transformation matrix from individual to the average brain to transform the ROIs back into each participant’s individual space. The motion corrected, smoothed and filtered data across each entire ROI were probed for %SC (i.e., individual loads as compared to resting baseline) for use in correlation analyses.

## Results

### Analysis I: Behavioral Performance

**Figure 2:**
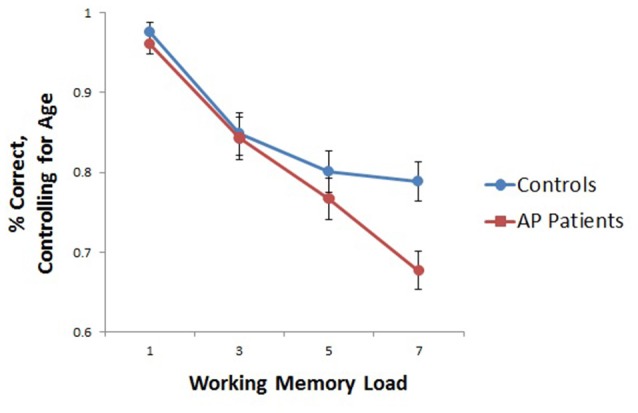
Group differences in working memory (WM)-task accuracy. As depicted, when adjusting for age, group differences in performance were significant at load seven only (*t*_(32)_ = −3.051, *p* < 0.01), although controls performed nominally better than adolescent psychosis (AP) patients at each WM load.

Age was significantly correlated with task performance (percent correct; *r* = 0.336, *p* < 0.001), and was thus included as a covariate in subsequent behavioral analyses. Because gender and task performance were not significantly correlated (*r* = −0.140, *p* = 0.429), gender was not included in final models. A repeated measures ANOVA showed a significant age-by-load interaction (*F*_(3,96)_ = 7.07, *p* < 0.001) along with a significant group-by-load interaction (*F*_(3,96)_ = 2.72, *p* < 0.05). Decomposed effects revealed that age was significantly positively correlated with increased task performance at load 3 only (*r* = 0.581, *p* < 0.001), and while controls performed nominally better than AP patients at each load, group differences in performance were significant at the highest load (Load 7) only (*t*_(32)_ = −3.051, *p* < 0.01; Figure 2). Reaction time did not significantly differ between groups (*t*_(32)_ = −0.08, *p* = 0.94).

### Analysis IIa: WM Capacity-Associated Neural Activity

**Figure 3:**
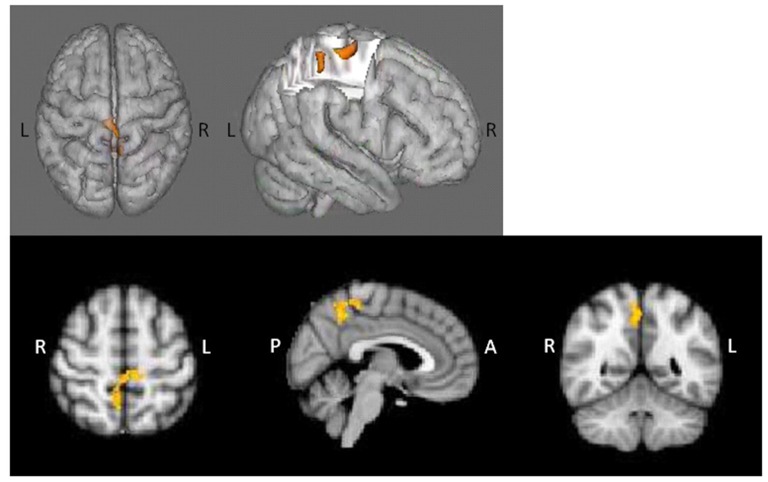
Main effect of group in whole-brain WM capacity analysis. Healthy controls displayed greater bilateral precentral and right postcentral/precuneus activity relative to AP patients.

AP patients evidenced lower overall WM capacity as compared to controls (*t*_(26)_ = 2.508, *p* < 0.05). Whole-brain analyses based on individual subject capacity revealed a significant main effect of group, with greater bilateral precentral and right postcentral gyrus and precuneus activity in healthy controls relative to patients (Figure 3). AP patients did not exhibit greater neural activity in any regions relative to controls.

### Analysis IIb: Age-Associated Effects on Neural Activity during Spatial Working Memory

**Figure 4:**
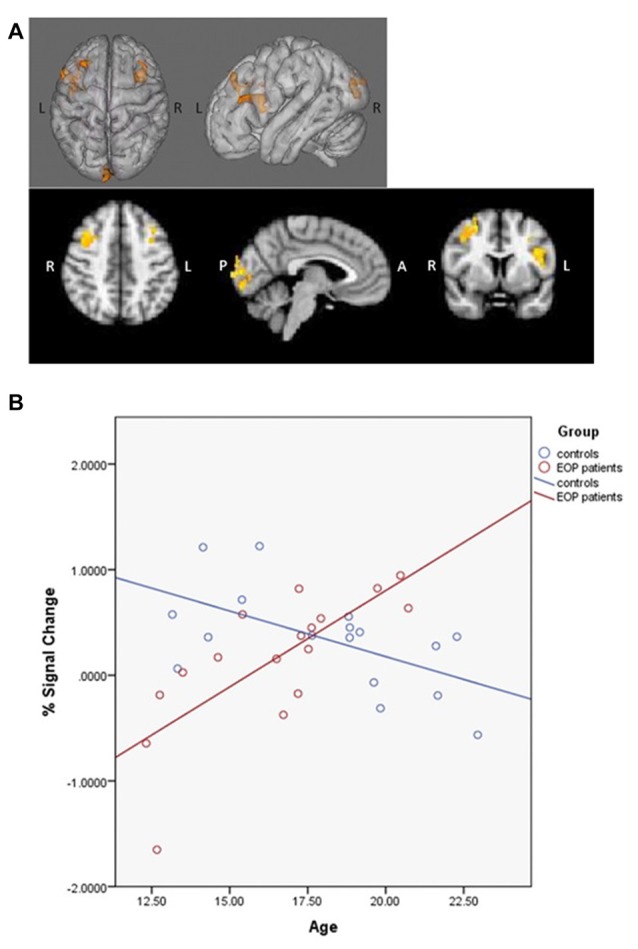
SCAP group-by-age interaction. Top panels **(A)** depict group differences in neural activity as a function of age, based on individual capacity. As shown, increasing age in the AP patients was associated with greater activity in bilateral middle frontal gyrus, right superior frontal gyrus, left inferior frontal gyrus, left insula, left lingual gyrus, left precentral gyrus and left occipital pole activation during task performance, which was not observed in healthy controls. The bottom panel **(B)** depicts the direction of effect based on percent signal change from the most significant cluster. While increased age was associated with increased task-based neural activity among AP patients, the opposite effect was observed among controls.

fMRI contrasts based on individual capacity also revealed a significant group-by-age interaction, with differentially greater activation in the bilateral middle frontal, right superior frontal gyrus, left inferior frontal gyrus, left insula, left lingual gyrus, left precentral gyrus and left occipital pole as a function of increasing age in AP patients relative to controls (Figure 4A); controls exhibited concomitant decreased activation in these areas (Figure 4B). Additionally, significant main effects of age indicated that, overall, older subjects exhibited greater activity in the left superior parietal lobule, precuneus, postcentral gyrus and lateral occipital cortex.

### Analysis IIIa: PPI Analysis

PPI analyses based on individual capacity revealed that, relative to youth with AP, controls exhibited greater connectivity between the left dlPFC and left frontal pole. No significant group differences were observed for any other ROI, and AP patients did not evidence greater co-activation between any regions as compared to healthy controls.

### Analysis IIIb: Age-Associated Effects on Connectivity

**Figure 5:**
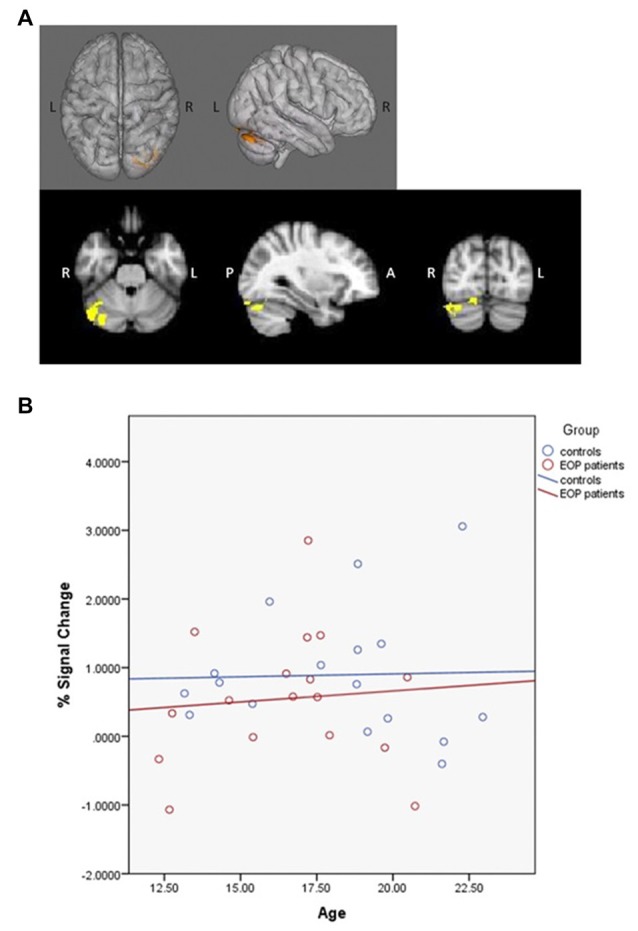
Psychophysiological interaction (PPI) group-by-age interaction for the left dlPFC. Top panels **(A)** depict that as compared to healthy controls, increased age among AP patients was associated with increased coupling between the left dlPFC and the right cerebellum, right lateral occipital cortex and right occipital fusiform gyrus. The bottom panel **(B)** depicts the direction of effect based on percent signal change from the single significant contrast cluster. While task-based neural activity did not vary with age among controls, increased age among AP patients was associated with an increase in activity.

PPI contrasts revealed a significant group-by-age interaction for the left dlPFC ROI (Figure 5). Increased WM-associated coupling between the left dlPFC and right cerebellum, right lateral occipital cortex and right occipital fusiform gyrus was observed among older as compared to younger AP patients; this pattern was not observed among healthy controls. All other age main effect and interaction contrasts were not significant.

### Analysis IVa: Association of Neural Activity with Task Performance

**Figure 6:**
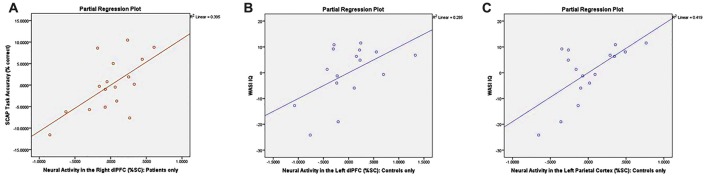
Correlation plots between neural activity and task performance/neurocognitive measures. Graphs depict **(A)** significant association between %SC in the right dlPFC and SCAP task accuracy among patients only (*r* = 0.628, *p* < 0.05); **(B)** significant association between %SC in the left dlPFC and IQ in control group only (*r* = 0.534, *p* < 0.05); and **(C)** significant association between %SC in the left parietal cortex and IQ among controls only (*r* = 0.648, *p* < 0.05).

Partial correlations controlling for the effects of age and gender revealed a significant relationship between right dlPFC activity and overall % correct among AP patients (*r* = 0.628, *p* < 0.05; see Figure 6A) but not among healthy controls (*r* = 0.146, *p* = 0.605). Correlations between task accuracy and %SC in all other ROIs were nonsignificant across both participant groups.

### Analysis IVb: Association of Neural Activity with Neurocognitive Measures

Controlling for the effects of age and gender, controls demonstrated a significant association between %SC in the left dlPFC and IQ (*r* = 0.534, *p* < 0.05; see Figure 6B), which was not observed in AP patients (*r* = −0.258, *p* = 0.373). Partial correlations also revealed a significant relationship between %SC in the left parietal cortex and IQ in controls (*r* = 0.648, *p* < 0.05; see Figure 6C), but not in AP patients (*r* = −0.356, *p* = 0.212). All other correlations between neuropsychological measures and %SC were not significant.

## Conclusion

This study investigated the nature and magnitude of spatial WM-related neural circuitry disruption as well as age-associated changes in WM circuitry in AP patients as compared to healthy adolescents. It further examined whether alterations in neural activity were related to neurocognitive functioning. To our knowledge, this is the first study to investigate both individual differences in WM capacity in AP and their relationship to development. The study yielded several main findings: (1) AP patients, relative to healthy controls, exhibited lower neural activity within bilateral precentral and right postcentral/precuneus areas during spatial WM performance when controlling for individual capacity, which is consistent with previous findings that did not incorporate capacity estimations (White et al., 2011a); (2) similarly, relative to typically developing controls, AP patients showed reduced coupling between the left dlPFC and frontal pole during WM task engagement relative to controls, which is distinct from a prior study suggesting reduced connectivity between the dlPFC and ACC, inferior parietal lobule and middle occipital gyrus among AP patients (Kyriakopoulos et al., 2012); (3) differential effects of age on neural activity and functional connectivity, respectively, suggest preliminary (cross-sectional) evidence for altered developmental trajectories in WM circuitry in AP; and (4) AP patients evidenced distinct relationships between neural activity and both SCAP task performance and global cognition as compared to controls, in that only patients showed an association between task accuracy and % signal change in the right dlPFC, whereas only controls demonstrated a significant association between IQ and % signal change in the left dlPFC and left parietal cortex.

Consistent with our hypotheses and with previous literature that both did (e.g., Bittner et al., 2015) and did not take individual capacity into account (White et al., 2011a), AP patients in the current study evidenced lower whole-brain activation in specific frontal and parietal regions relative to healthy adolescents during a WM task. However, we identified fewer regions of significant group differences in neural activity as compared to other reports that did not factor in individual capacity differences (Kyriakopoulos et al., 2012). We did not find evidence of hyperactivation in prefrontal and temporal regions in AP patients relative to controls, which has been reported in some prior studies of youth with AP that did not include capacity and utilized either an n-back (Thormodsen et al., 2011; Sugranyes et al., 2012) or Sternberg paradigm (White et al., 2011a). Some of these distinctions may be accounted for by paradigm differences, particularly those that primarily utilized verbal WM tasks vs. our spatial WM design (e.g., Kyriakopoulos et al., 2012; Sugranyes et al., 2012). As previously suggested, recent work points to a generalized inefficiency of WM circuitry that varies by WM load (Potkin et al., 2009). Thus, discrepancies in prior neural findings may be reflective of how well the capacity of each participant mapped on to the various task demands, which has not been well considered to date.

Controlling for individual capacity may have also led to distinct patterns of functional connectivity, indicating greater co-activation between the left dlPFC and left frontal pole among controls, relative to AP patients. This suggests that at their own maximum WM level, controls are better able to sustain the prefrontal network to process visual information as compared to patients. This is consistent with previous PPI work among healthy adults showing that increased connectivity between bilateral frontoparietal areas, as a function of increasing WM load, predicted better n-back task performance (Cassidy et al., 2016). Although the PPI approach has not been widely applied to the SZ WM literature, previous findings in an AP sample similarly noted reductions in dlPFC coupling, albeit with other brain regions (ACC, occipital gyrus and inferior parietal lobule; Kyriakopoulos et al., 2012). However, in addition to not accounting for capacity, Kyriakopoulos et al. (2012) utilized a letter-based 2-back task that did not parametrically vary WM demand, perhaps also accounting for the lack of performance deficit in the SZ group that we and others have found.

This study additionally found a positive association between age and frontal and occipital activation at WM capacity in individuals with AP. In contrast, among healthy controls, WM-related brain activity in some of these regions (e.g., right superior frontal gyrus) has instead been shown to negatively correlate with age (Andre et al., 2016). Previous work has identified a progression of increasing network specialization from childhood to adulthood, in that children are more likely to recruit regions such as the lateral cerebellum and thalamus, while adolescents rely on premotor and inferior parietal regions, and adults primarily recruit the dlPFC and ventromedial PFC (Klingberg et al., 2002; Scherf et al., 2006; Geier et al., 2009). Cerebellar recruitment during visuospatial WM tasks has been uniquely found among children, and has been associated with unskilled performance related to error detection and corrections (Scherf et al., 2006). Here, AP patients also demonstrated increased coupling between prefrontal and occipital/cerebellar regions with increasing age, suggesting more pronounced network inefficiency over time. Thus, AP patients evidence atypical development of WM-related regions, consistent with our hypotheses. Results are also in line with previous findings suggesting differential recruitment of cerebellar regions among patients with SZ as compared to healthy controls during WM tasks (Meyer-Lindenberg et al., 2001).

These functional findings are corroborated by behavioral and WM capacity group differences. Patients performed with decreased task accuracy as compared to controls, significantly so at the highest WM demand, which is in agreement with our hypotheses and previous literature (Lee and Park, 2005; Piskulic et al., 2007; White et al., 2011a; Bittner et al., 2015). Correspondingly, patients evidenced reduced overall WM capacity compared to controls, thus leading to expectations that their performance would degrade accordingly above that lowered threshold. Task accuracy also correlated with neural activity in the right dlPFC among patients only, suggesting atypical recruitment of frontal regions while attempting to sustain performance. Of note, dlPFC activity has been shown to increase parametrically with WM demand until load exceeds the individual’s capacity, though WM capacity for those with SZ is reduced relative to controls (Manoach, 2003). Given our study’s selection of each individual’s optimal load/capacity, it is possible that findings reflect patient’s experience of a more challenging task relative to controls, thus requiring increased dlPFC recruitment to sustain better task accuracy. In fact, previous literature has also suggested that increased task difficulty via increased WM demand correlates with increased frontal lobe activation, as well as decreased activation in visually-mediated areas (e.g., Grady et al., 1996; Bokde et al., 2005; Höller-Wallscheid et al., 2017; Siciliano et al., 2017), comparable to our study findings.

Higher neural activity in both the left dlPFC and left parietal regions was associated with higher overall intelligence among healthy controls only. This suggests that neural activity during higher-order cognitive tasks is less predictive of global cognition in AP patients relative to healthy controls. While prior research among adults with SZ has demonstrated positive correlations between cognitive functioning and WM/capacity (Piskulic et al., 2007; Gold et al., 2010; Johnson et al., 2013), the relationship may be attenuated as compared to healthy individuals (Gold et al., 2010). Prior work has suggested that the neural mechanisms leading to reduced WM capacity in SZ are not identical to those producing variations among healthy controls (Vogel and Machizawa, 2004; Gold et al., 2006; Leonard et al., 2013); for AP patients who are undergoing atypical neural development of WM-related networks, these correlations may be even more diminished when compared to typical adolescents.

It is important to note that the regression analyses examining relationships between neural activity, task performance and neurocognitive measures were exploratory and would not survive corrections for multiple comparisons. This is likely due in large part to the limitations of our sample size and the heterogeneity of the patient sample, including the wide age range of participants. Future, larger-scale studies may benefit from conducting analyses with subjects stratified by age clusters. Moreover, we were unable to investigate effects of age of illness onset on neural and behavioral WM measures; however, earlier onset may yield more significant impairment across multiple cognitive domains as compared to adult-onset patients (Basso et al., 1997; Collinson et al., 2003; Rajji et al., 2009; Frangou, 2010). Furthermore, given the extensive history of psychotropic medication use in several patients, studies with medication-naïve AP individuals would be necessary to confirm that observed differences were independent of medication effects. Lastly, an important caveat of this version of the SCAP task (Glahn et al., 2003) is that the maintenance period always follows the encoding period; as jittering was not utilized between trials, BOLD signal from the encoding period may contaminate the signal within the maintenance period. However, as only correct response trials were modeled in analyses, the interference of encoding on the maintenance signal may be relatively minimized.

Through emphasizing early indicators of neural dysfunction, this work has the potential to better elucidate endophenotypes of SZ (Glahn et al., 2003; Wood et al., 2003). The abnormal developmental trajectories of WM-associated neural activity that we observed in youth with AP also suggest a window of opportunity for early intervention. Visual WM capacity in both healthy adults and adult patients with SZ is strongly correlated with overall cognitive abilities (Kyllonen and Christal, 1990; Johnson et al., 2013; Luck and Vogel, 2013). Replication of this work in AP samples is critical to determine if reduced capacity can lead to decreased intellectual functioning over time (Luck and Vogel, 2013). Studies have demonstrated that early detection and treatment of SZ is associated with improved long-term outcomes (Larsen et al., 2011). These findings suggest reduced WM capacity may be a key area for potential cognitive remediation studies. Finally, this work further highlights the need for longitudinal studies, which are essential to determine when in the course of development abnormal patterns of WM-associated neural activity emerge.

## Author Contributions

AE conceptualized, planned and executed the analyses, interpreted the data and wrote the first draft of the manuscript. KHK assisted in planning, execution of analyses, interpretation and manuscript editing. KMH aided in data interpretation and generation of the manuscript. PB and MJ aided in data collection and manuscript preparation. JZ provided administrative and clinical support for the study. TGME supported data collection and manuscript writing. TDC provided the environment for which the study protocol was carried out and designed and contributed to manuscript preparation. CEB supervised the current project and had roles in conceptualization, planning, analysis execution, data interpretation and manuscript writing. All authors contributed to and gave approval to the manuscript.

## Conflict of Interest Statement

The authors declare that the research was conducted in the absence of any commercial or financial relationships that could be construed as a potential conflict of interest.

## Footnotes

^1^http://fsl.fmrib.ox.ac.uk/fsl/fslwiki/FSLMotionOutliers

^2^https://fsl.fmrib.ox.ac.uk/fsl/fslwiki/FEAT/UserGuide#Featquery_-_FEAT_Results_Interrogation
